# The Microbiome in Inflammatory Bowel Disease

**DOI:** 10.3390/jcm13164622

**Published:** 2024-08-07

**Authors:** Aranzazu Jauregui-Amezaga, Annemieke Smet

**Affiliations:** 1 Department of Gastroenterology and Hepatology, University Hospital Antwerp, 2650 Edegem, Belgium; 2 Laboratory of Experimental Medicine and Pediatrics (LEMP), Faculty of Medicine and Health Sciences, University of Antwerp, 2610 Wilrijk, Belgium

**Keywords:** microbiota, Crohn’s disease, ulcerative colitis, biomarker, biofilm, dysbiosis

## Abstract

The management of patients with inflammatory bowel disease (IBD) aims to control inflammation through the use of immunosuppressive treatments that target various points in the inflammatory cascade. However, the efficacy of these therapies in the long term is limited, and they often are associated with severe side effects. Although the pathophysiology of the disease is not completely understood, IBD is regarded as a multifactorial disease that occurs due to an inappropriate immune response in genetically susceptible individuals. The gut microbiome is considered one of the main actors in the development of IBD. Gut dysbiosis, characterised by significant changes in the composition and functionality of the gut microbiota, often leads to a reduction in bacterial diversity and anti-inflammatory anaerobic bacteria. At the same time, bacteria with pro-inflammatory potential increase. Although changes in microbiome composition upon biological agent usage have been observed, their role as biomarkers is still unclear. While most studies on IBD focus on the intestinal bacterial population, recent studies have highlighted the importance of other microbial populations, such as viruses and fungi, in gut dysbiosis. In order to modulate the aberrant immune response in patients with IBD, researchers have developed therapies that target different players in the gut microbiome. These innovative approaches hold promise for the future of IBD treatment, although safety concerns are the main limitations, as their effects on humans remain unknown.

## 1. Introduction

Inflammatory bowel disease (IBD), including Crohn’s disease (CD) and ulcerative colitis (UC), is a chronic inflammatory condition that primarily affects the gastrointestinal tract. The aetiopathogenesis of the disease has not yet been clarified, but it is believed that an abnormal immune response is triggered by an imbalanced gut microbiome, due to an unknown factor. Various complex interactions occur between the different factors involved, such as the intestinal microbiome, the innate and adaptive immune systems, soluble mediators, and host cells [1]. It is thought that the balance between bacterial antigens and the host’s immune system is lost, leading to a disruption in the mucosal barrier and the translocation of antigens to the lamina propria, which triggers acute and chronic inflammatory immune responses.

## 2. Main Concepts Associated with the Human Gastrointestinal Microbiome

### 2.1. Microbiota and Microbiome

Research on IBD has traditionally focused on gut bacteria due to technical limitations [2]. However, other intestinal microorganisms, such as fungi, protozoa, viruses, and bacteriophages, may be involved in the pathogenesis of IBD [3].

The microbiota is a collection of microorganisms, including prokaryotes (bacteria and archaea), eukaryotes (microbial parasites and fungi), and viruses, found in a specified environment [4]. In the human gastrointestinal tract, the microbiota comprises all of these microbial communities from the mouth to the anus. It is established early in life and becomes stable in the first 2 to 3 years of life. Although it is a dynamic ecosystem, it maintains functional stability to perform its main functions: (a) barrier function, limiting the establishment of pathogenic bacteria via competition for nutrients and the synthesis of antimicrobial peptides; (b) nutrition, metabolising and synthesising nutrients; and (c) interaction with the immune system for its development and maturation. The microbiota composition is influenced by various factors and changes along the gastrointestinal tract, being low at the upper gastrointestinal tract and increasing progressively towards the colon, where it reaches its highest concentration and diversity. The luminal load of bacteria in the colon can reach more than 10^14^ per gram of colonic content [5,6].

The term microbiome implies a broader concept that includes the microbiota, the genes and gene products of the microbiota, and their surrounding microenvironment [7]. The microbiome interacts with the host through metabolites; some of them are diet-derived metabolites and others are synthesised de novo by microorganisms. The main metabolic pathways that are thought to be involved in gut homeostasis are short-chain fatty acid (SCFA) production, bile acid metabolism, and tryptophan metabolism.

The mycobiome or fungal microbiome includes the fungal community of a specific environment with its genetic and environmental information. Similarly, viruses can be referred to as the virome. There is still a lack of accurate information on these populations due to limitations in culture and sequencing techniques. The most abundant phyla of the intestinal fungal microbiome are dominated by Ascomycota and Basidiomycota, and the most frequent species are yeasts such as *Candida*, *Malassezia* and *Saccharomyces* [8,9]. Regarding the intestinal virome, it is divided into two subtypes. The first type is the virus that infects eukaryotic cells, such as human cells. The second is the phages that infect bacteria, also called bacteriophages, and they represent most of the viral species in the gut. Given that bacteriophages can transfer genetic content between bacterial cells, they have a relevant role in intestinal infections: they can transfer antibiotic resistance genes between bacterial cells or cause the quick destruction of bacterial cells upon infection during the lytic cycle. Consequently, these viruses can regulate the population levels of resident bacteria. The human gut phage population, or phageome, is composed of either DNA or RNA. *Caudovirales* and *Microviridae* are the main members of the human gut phage community, with *Caudovirales* being the predominant phage [10].

Archaea are single-celled microorganisms with a structure similar to bacteria. They are believed to constitute an ancient group that is intermediate between bacteria and eukaryotes and are also present in the human gut microbiome. The most frequently identified is *Methanobrevibacter smithii*, a methane-producing archaeon.

**Figure 1:**
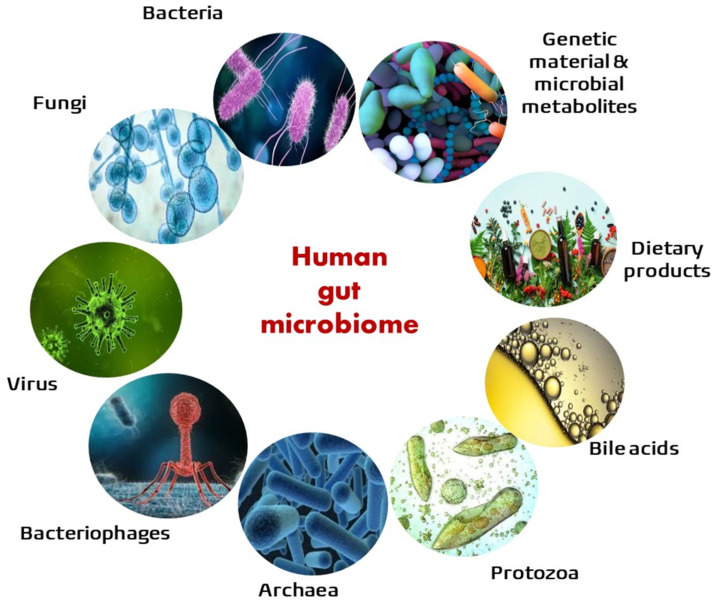
Schematic representation of the human intestinal microbiome. The microbiota is a broad concept that includes microorganisms (the microbiota), the genes and gene products of the microbiota, and the microenvironment. Most nutrients are digested and absorbed in the small intestine. However, dietary fibre remains intact until it reaches the colon. In the colon, a fermentation process is performed by enzymes produced by gut bacteria, resulting in short-chain fatty acid (SCFA) production, including acetate, propionate, and butyrate. These metabolites participate in various cellular and immunological processes. They stimulate the production of mucins, reduce the intestinal permeability, and promote anti-inflammatory pathways. Among the SCFAs, butyrate is the main energy source for the intestinal epithelial cells and has modulator functions that lead to a decreased concentration of oxygen in the intestinal lumen. As a result, the number of obligate anaerobic bacteria, including those of the phylum Firmicutes, which produce butyrate, increases. Bile acids (BAs) are the end products of cholesterol catabolism and are released into the small intestine through the ampulla of Vater. They form micelles with lipid molecules and facilitate their absorption in the small bowel through the enterohepatic circulation. However, there is a small proportion of BAs that remain in the gut and is metabolised by the gut bacteria [11]. Tryptophan is an essential amino acid that should be ingested with the diet. Gut bacteria convert it into tryptamine and other products. These products can function as endogenous ligands for the aryl hydrocarbon receptor (AhR), an essential signalling pathway in the maintenance of gut homeostasis [12].

Research on the microbiome has evolved from characterising community compositions to a more holistic approach that seeks to understand the dynamic interactions between all constituents of the microbiome and the host over time, in both health and disease. In Figure 1, all of these terms are represented.

### 2.2. Mucosal Barrier and Mucins

The mucus barrier creates a protective layer that covers the intestinal epithelial surface. It is one of the first lines of defence in the gastrointestinal tract against external substances, digestive enzymes, and microorganisms. The mucus layer serves as a diffusion barrier, allowing small molecules such as ions, water, nutrients, and gases to reach the enterocytes. Additionally, it is part of the innate mucosal intestinal barrier and acts as the first line of immunological defence. Secreted and transmembrane mucins represent the major components of the mucus barrier [13]. Besides having a protective function, transmembrane mucins also participate in intracellular signal transduction and play an essential role in epithelial cell homeostasis by modulating junctional protein expression [13,14].

**Figure 2:**
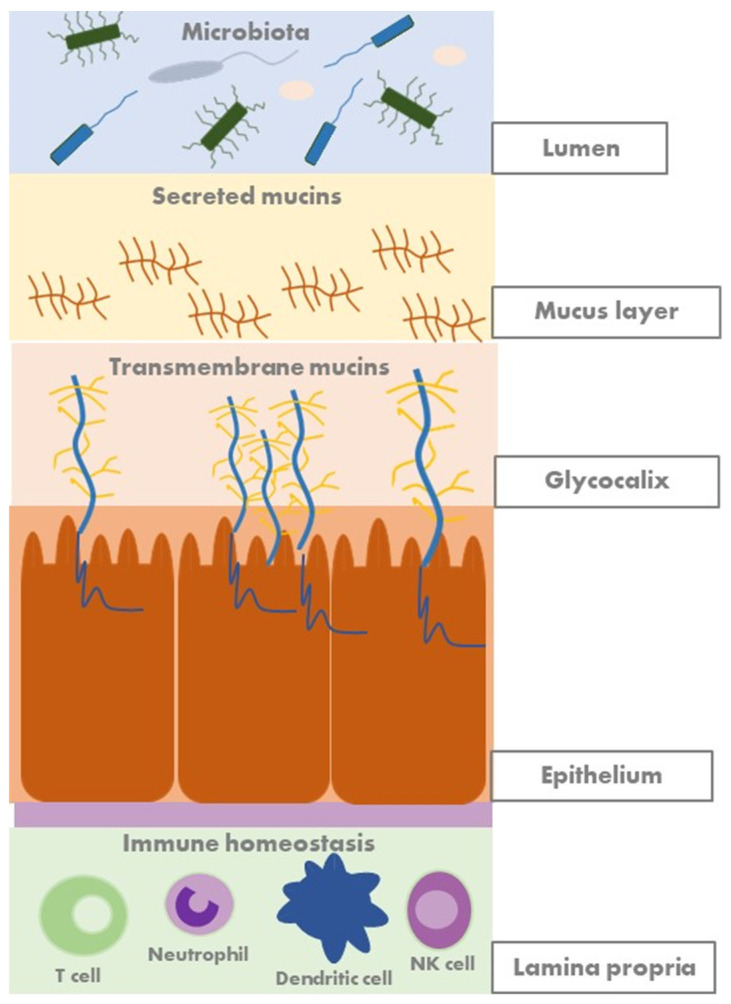
Overview of the intestinal mucosal barrier. The human gut is a vast surface of contact with the environment that is colonised by trillions of gut microbes. The intestinal barrier comprises a thick layer of mucus, a single layer of epithelial cells, and the inner lamina propria hosting innate and adaptive immune cells. The intestinal epithelium, along with the mucus layer that covers it, acts as a physical barrier.

Furthermore, a recent study has shown that the intestinal aberrant mucin mRNA expression levels are associated with IBD presentation and activity, highlighting their potential as biomarkers to monitor mucosal barrier function in IBD [15]. Mucins have also immunological effects, binding directly to immune cells [14], and play a crucial role in interacting with the gut microbiota, providing nutrients and attachment sites. Figure 2 shows a schematic representation of the human gut mucosal barrier.

### 2.3. Methods for the Analysis of the Microbiome: Multi-Omics

Research on the gastrointestinal microbiome has long been limited due to inadequate technical methods. However, with the development of new sequencing techniques in recent years, the complexity and diversity of the human gastrointestinal microbiome are being revealed [16,17].

When analysing the microbiome, there are two main types of gut samples: stool samples and biopsy samples. Stool samples examine the luminal content, while ileal or colonic mucosal biopsies evaluate the mucosa-associated microbiome. There are differences in the microbial composition between faecal and mucosal samples [18]. Most bacteria are believed to be tightly adhered to the mucus layer. In the same way, the sampling site while taking mucosal biopsies in patients with IBD may show different results, based on the presence or absence of inflammation (inflamed versus non-inflamed tissue) [18].

**Figure 3:**
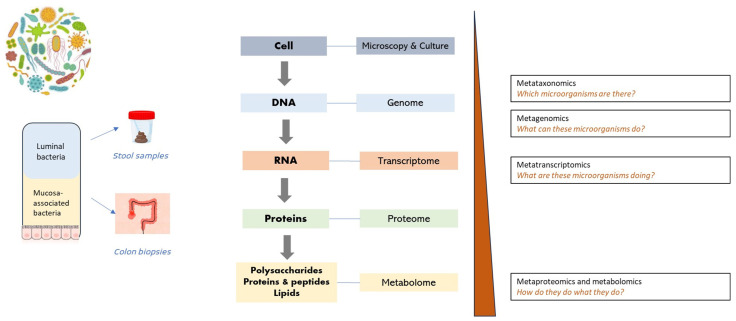
Multi-omics for the study of the human gut microbiome in inflammatory bowel disease. Metagenomics involves identifying the bacterial composition and diversity using techniques such as 16S rRNA gene amplicon or shotgun sequencing. This method provides information on the presence or absence of specific genes in the microbiome. Metatranscriptomics focuses on assessing the functionality of the microbiome by analysing gene expression over time using RNA sequencing techniques. Proteomics studies the entire set of proteins that a genome can express in a cell, known as the proteome. Combining metagenomic and metaproteomic data, it is possible to characterise signalling proteins and pathways. Metabolomics explores the metabolome, which consists of metabolites derived from both the host and microorganisms.

For decades, microbiology has relied on culture-dependent methods to identify bacteria involved in human health or disease. Culture-dependent methods involve the cultivation of microorganisms in the laboratory and the determination of the viable population of microorganisms. These methods enable the classification of bacteria to the species and strain level. However, only a fraction of the microbial populations can be identified, due to the bias towards bacteria that proliferate under laboratory conditions. Nonetheless, the development of molecular diagnostic techniques, mainly next-generation sequencing (NGS) techniques, represents a breakthrough in understanding the human gastrointestinal microbiome, enabling the discovery and characterisation of unculturable microorganisms and the prediction of their functions [19,20]. Figure 3 summarises the multi-omics techniques that can be used for the study of the human gut microbiome in IBD.

## 3. Changes in the Microbiome in Patients with Inflammatory Bowel Disease

### 3.1. Gut Dysbiosis

The human gut microbiome maintains a mutually beneficial relationship with the colonised host. It is key in maintaining health by metabolising dietary components, producing essential components such as vitamin K and SCFAs, and assisting in the development and function of the immune system [21].

The incidence of IBD is on the rise in newly industrialised countries, which is believed to be associated with a Western lifestyle, urbanisation, and industrialisation. The “hygiene hypothesis” suggests that there is a link between the increasing incidence of autoimmune and allergic diseases in industrialised nations and the improvements in sanitation and hygiene conditions [22]. This situation would limit the exposure to microorganisms, leading to an impaired immune response later in life.

In the healthy population, there is a balance of bacterial species in the gut. More than 90% of the healthy bacterial species in the human gut microbiome belong to four major phyla: Bacteroidetes, Firmicutes, Actinobacteria, and Proteobacteria. However, there is a significant inter-individual microbial diversity difference (beta diversity) within these major phylotypes [23].

The loss of this balance leads to gut dysbiosis, which is considered to be one of the triggers of an inappropriate immune response in the development of IBD [24]. Studies show differences in the abundance of some intestinal bacteria in patients with IBD as compared to controls. Gut dysbiosis is characterised by an increase in the number of mucosa-associated bacteria and a reduction in overall biodiversity. In patients with IBD, there is a decrease in beneficial bacteria, such as those of the phylum Firmicutes, of which the most studied is *Faecalibacterium prausnitzii* [25]. On the other hand, members of the phylum Enterobacteriaceae, which includes *Escherichia coli*, are found to be increased in IBD and are thought to play a pathogenic role in the development of the disease. Changes in *Bacteroides* spp., particularly in patients with CD, have also been observed [26].

Until recently, faecal samples were commonly used to evaluate the gut microbiome and its alpha diversity, which measures the within-individual composition of the gut microbiome. It was thought that the alpha diversity was high in healthy individuals and reduced in patients with IBD. However, advancements in technical methods have enabled the study of the mucosal-associated microbiome, revealing lower alpha diversity and a lower abundance of most microbes in healthy individuals [27].

Although other members of the gut microbiome, such as fungi and viruses, have been less investigated, some data suggest their implication in IBD pathogenesis. Studies on the fungal population in patients with IBD have shown controversial results. One hypothesis is that intestinal inflammation results in a compromised mucosal barrier that allows the proliferation of opportunistic fungi that interfere with the host immune system. Compared with healthy individuals, an increased proportion of *Candida albicans* was observed [9]. However, data showing no pathogenic role of fungi have also been published [8]. The abundance change in gut fungi is not only restricted to the faecal microbiome but also occurs in the inflamed mucosa [28]. Gut dysbiosis also affects the compositions of the virome and phaegome. While the faecal and mucosal virome remains unclear in both health and IBD, a recent study explored the composition and functionality of the ileal mucosal virome in healthy adults and in patients with CD [29]. There was substantial depletion in the richness of bacteriophages, whereas the richness of eukaryotic viruses was increased, suggesting that bacteriophages and eukaryotic viruses may have discrepant roles in the pathogenesis of the disease.

The metabolic pathways of the gut microbiome are also altered in IBD. It is believed that a decrease in SCFAs, and particularly a decrease in butyrate-producing bacteria and the concentration of butyrate, may be involved in the development and persistence of chronic intestinal inflammation in IBD. In the same way, metabolomic studies have revealed disturbances in BA metabolism in patients with IBD, with an increase in primary BAs and a reduction in secondary BAs [11]. Regarding tryptophan metabolism, there is decreased production of AhR ligands in the microbiota of patients with IBD, and the AhR expression in intestinal tissue can be decreased [30,31].

Unfortunately, it is difficult to determine whether the changes in the microbiome are the cause or the consequence of inflammation. However, differences in the gut microbiota composition have been observed in members of the same family, and even between twins. This suggests that gut dysbiosis in IBD is more associated with the disease state than with genetic or environmental factors [32]. Chronic inflammation may boost dysbiosis through the metabolic and oxidative alteration of the intestinal environment [33]. There are also intra-individual microbiota changes indicative of disease activity. The alterations in the faecal microbiome composition are most marked in an active disease, particularly in CD [34].

### 3.2. Microbiota as a Biomarker

The term “biomarker” refers to a characteristic that is objectively measured and evaluated as an indicator of normal biological processes, pathological processes, or pharmacological responses to a therapeutic intervention [35]. The gut microbiome has been evaluated as a biomarker for the diagnosis and monitoring of patients with IBD.

In a study conducted on children, microbiome analysis was used as a non-invasive diagnostic technique to identify patients with suspected IBD [36]. This helped to select those who required endoscopy for diagnosis confirmation [36]. A systematic review and meta-analysis also observed that gut dysbiosis in newly diagnosed treatment-naïve patients resulted in reduced microbial abundance, less biodiversity in the structure of microbial communities, and differential bacterial abundances compared to the profiles of established and treated patients or control groups [18].

Furthermore, the composition and functionality of the microbiome can also be modified by IBD treatment. The use of biological agents such as anti-TNF, anti-integrins, or anti-interleukins has been observed to increase the diversity and promote recovery from intestinal eubiosis after administration in patients with CD [37]. This led to a relative increase in SCFA-producing microorganisms, such as Firmicutes and Bacteroidetes, and a decrease in the relative abundance of Proteobacteria. Therefore, changes in the gut microbiome suggest its potential application as a biomarker to predict the treatment response in patients with IBD.

The microbiota of patients with IBD in remission who discontinue anti-TNF therapy has also been analysed. The results showed that patients who remained in remission over time had a significant increase in the phyla Bacteroidetes and Firmicutes. Although there is less scientific evidence with vedolizumab and ustekinumab, the results are in the same direction [37].

Gut microbiome analysis was also helpful in differentiating patients with IBD from patients with irritable bowel syndrome [38]. Although there were similarities between these two populations compared to control individuals, the gut microbiota composition was able to distinguish patients with IBD from those with irritable bowel syndrome.

However, the applicability of the gut microbiome as biomarker for the management of IBD is limited due to its overlap with that of normal subjects, as well as its heterogenicity prior to disease onset and during IBD evolution [39].

## 4. Modulation of the Gut Microbiome in Inflammatory Bowel Disease

The goal in the management of patients with IBD is to achieve mucosal healing, which involves restoring the barrier function of the intestinal epithelium [40]. The current therapies aim to reduce the aberrant immune response based on the degree of activity, location, and behaviour of the disease [41,42,43,44]. Various approaches have been proposed to modulate the gut microbiome and expand the therapeutic options beyond standard medical therapies.

### 4.1. Early-Life Exposure

The development of the gut microbiome begins at birth and involves the acquisition of bacterial communities from the mother. There is a gradual diversification of the gut microbiome during this process, which can be modulated by different environmental factors. The early stages of life represent a crucial opportunity to modulate the gut microbiome and immune function, which can have a significant impact on health and disease later in life [45].

Potential interventions that may reduce the risk of developing IBD during delivery and early life include vaginal childbirth, breastfeeding, minimising the use of antibiotics, avoiding exposure to tobacco smoke, maintaining a healthy diet, and exposure to nature [6].

Studies have shown that the increase in the number of caesarean sections runs parallel to the increase in IBD cases. Newborns born by caesarean section have a skin-type microbiota, whereas vaginal newborns acquire the mother’s vaginal microbiota. Breastfeeding is associated with a lower incidence of IBD in children. Exposure to tobacco smoke has been identified as a risk factor for the development of CD, but not UC. Patients who quit smoking have an increased risk of UC, showing an inverse relationship. The incidence of UC is lower in patients who have undergone an appendectomy. The use of medications such as antibiotics or non-steroidal anti-inflammatory drugs has been associated with IBD. Additionally, oral contraceptives have been found to increase the risk of CD. Vitamin D deficiency is common in patients with newly diagnosed IBD and has been linked to an increased risk of CD. Studies have shown that stress, anxiety, and depression increase the risk of IBD, while exercise reduces the risk. Regarding vaccines, there is no evidence to support that vaccinations administered during childhood can lead to the subsequent development of IBD.

### 4.2. Physical Activity

Growing evidence suggests that physical activity has an impact on the gut microbiome [46]. Clinical interventions have shown that physical activity can affect the gut microbial diversity and cause significant changes in the abundance of specific gut bacterial groups and bacterial metabolites. However, there are limited data on the frequency, intensity, and types of physical activity needed to guide implementation. Additionally, the relationship between physical activity and the onset or natural history of IBD is not yet fully understood.

### 4.3. Diet

Diet has become a crucial aspect in the management of IBD. In children with active CD, exclusive enteral nutrition (EEN) can help to achieve remission rates of up to 80% [47]. However, in the adult IBD population, lower rates of efficacy, up to 45%, have been observed, which could be due to lower rates of treatment adherence [48]. While the mechanism of action is not fully understood, research suggests a paradoxical effect of EEN on the microbiome, inducing a reduction in bacterial diversity and richness [49,50]. Therefore, the anti-inflammatory effect of EEN is considered to be more complex, rather than the modulation of the gut microbiota. There are several hypotheses to explain the effect of EEN in children with CD, including decreased exposure to food antigens, exogenous bacteria, and/or bacterial components present in a regular diet, or a change in cytokine profiles [51,52].

The ”Crohn’s Disease Exclusion Diet” combined with partial enteral nutrition is the first dietary strategy involving the consumption of real food that provides convincing evidence of reduced inflammatory activity in CD [53]. Currently, there is no evidence to support the implementation of other predefined dietary regimens that involve the significant restriction or complete exclusion of one or more suspect food groups in adult patients with IBD [54,55,56]. While exclusion and elimination diets may improve symptoms in patients with IBD, they have no effect on objective markers of inflammation.

However, according to some epidemiological studies, consuming ultra-processed foods, food additives, and emulsifiers may increase the risk of developing IBD through various mechanisms. One theory suggests that consuming higher amounts of ultra-processed foods may result in lower fibre intake, normally present in minimally processed food. Another hypothesis links ultra-processed food with higher levels of salt and artificial sweeteners, which can cause intestinal inflammation [57,58].

It is still unknown whether the dietary components that may act as triggers at the onset of the disease are those that can worsen inflammation in patients with established CD. It is pointed out that EEN formulas are highly processed products that can vary significantly in terms of their macronutrient composition, non-nutrient components, and food additives [59]. Therefore, these dietary components are unlikely to be either the mechanisms that determine the success of EEN administration or significant CD triggers.

### 4.4. Antibiotics

Since the discovery of the first antibiotic in 1940, the use and spectrum of antimicrobial activity have increased steadily. However, antibiotics have side effects on the resident microbiota, inhibiting certain bacterial populations and enhancing others [60]. It has been observed that, after antibiotic use, it takes months for the flora to return to its pre-treatment state. Moreover, excessive and inappropriate antibiotic use can lead to not only antimicrobial-resistant infections but also the increased incidence of chronic non-communicable diseases. Antibiotic consumption during early life, coupled with genetic susceptibility, may lead to changes in the gut microbiota that generate chronic inflammatory changes in the immune system. This can ultimately result in the development of diseases, including IBD. Two large national database studies, one conducted in the UK involving over one million children and the other in Denmark with over half a million children, have revealed that antibiotic exposure during childhood is associated with an increased risk of developing IBD later in life [61,62]. This association was found to be dose-dependent, with infants who received antibiotics in the first year of life being at a higher risk than those who were not exposed to antibiotics.

On the other hand, the use of antibiotics for the management of IBD has been a topic of discussion. Some studies have suggested that antibiotics may have a modest beneficial effect on the control of IBD flares. However, recent systematic reviews conducted on both paediatric and adult populations have found no significant difference in the effectiveness of antibiotics compared to a placebo in treating CD and UC [63,64,65]. The only clear indication for antibiotic treatment in IBD is in managing acute pouchitis in both adults and children, beyond bacterial superinfections [43,66].

### 4.5. Probiotics

Probiotics are live microorganisms that, when administered in adequate amounts, confer a health benefit on the host [67]. The strains commonly used as probiotics are lactic acid bacteria and Bifidobacteria, which are isolated from traditional fermented products, as well as the human gut, faeces, and breast milk [68]. However, probiotics can also be isolated from the guts of various animal species.

Probiotic strains can impact the intestinal environment by influencing the mucosal immune mechanisms, interacting with commensal or potential pathogenic microbes, generating metabolic end products such as SCFAs, and communicating with host cells through chemical signalling. These mechanisms are believed to contribute to their anti-inflammatory properties, including the reduction of the incidence and severity of diarrhoea, which is one of the most widely recognised uses of probiotics.

The World Gastroenterology Organisation (WGO) has recently published new clinical guidelines that examine the use of probiotics and prebiotics in IBD [67]. According to the guidelines, probiotics can be useful in the management of pouchitis for both primary and secondary prevention. However, while some studies have shown that probiotics can help to induce remission in patients with CD or UC, there is not enough robust evidence from systematic reviews to recommend their use [69]. The benefits of probiotics in managing pouchitis in patients who have undergone a proctocolectomy for UC have been known for years. The European clinical guidelines recommend the use of a probiotic called *VSL#3*, which is a combination of eight probiotic strains, for this indication [43]. Additionally, the combination of *Saccharomyces boulardii* and *VSL#3* showed favourable results in patients with CD [70]. The European Society for Clinical Nutrition and Metabolism (ESPEN) recently updated its recommendations on IBD management and added a new chapter on microbiota modulation, specifically for children [66]. The updated guidelines suggest that probiotics can be used as an alternative to standard 5-aminosalicylic acid therapy in patients with UC, if the latter is not tolerated, for the treatment of mild to moderate active disease.

### 4.6. Prebiotics

Modulating the gut microbiota composition through diet manipulation, specifically through the use of prebiotics, has gained significant attention. Prebiotics are selectively fermented ingredients that result in specific changes in the composition and/or activity of the gastrointestinal microbiota, thus conferring benefits upon host health, such as a decrease in potentially pathogenic microorganisms or the potentially harmful metabolic activities of the microbiota [67].

Prebiotics typically consist of non-starch polysaccharides and oligosaccharides, although other substances, such as resistant starch, conjugated linoleic acid, and polyphenols, are being studied as candidate prebiotics. Most prebiotics are used as food ingredients. Some commonly known prebiotics are oligofructose (also known as fructooligosaccharide, FOS), inulin, galactooligosaccharides (GOSs), lactulose, and breast milk oligosaccharides (human milk oligosaccharides or HMOs). The use of prebiotics in patients with IBD is not yet well established. The results of a systematic review and meta-analysis showed that prebiotics in IBD patients were associated with a less inflammatory pattern, based on measurements of faecal calprotectin, the interleukin profile, a metabolome study, and the detection of the increased presence of bifidobacteria [71].

### 4.7. Postbiotics

In 2021, the International Scientific Association for Probiotics and Prebiotics (ISAPP) published a consensus document on postbiotics and defined them as preparations of inanimate microorganisms and/or their components that confer a health benefit to the host [72]. Short-chain fatty acids, which are a product of the interaction between the diet and gut microbiota, have been subjected to clinical trials in humans, and the results have been encouraging. For instance, in IBD clinical trials, butyrate enemas have been used to treat UC, and they have led to a modest improvement in symptoms [73].

### 4.8. Faecal Microbiota Transplantation

Faecal microbiota transplantation (FMT) is a different approach to managing gut dysbiosis. This procedure involves the infusion of faeces from healthy donors into the gastrointestinal tracts of recipients to treat disease-associated gut dysbiosis. The procedure was first used in 1958 as an enema to treat patients with life-threatening fulminant pseudomembranous enterocolitis, with good results [74]. However, the standardised use of FMT for the treatment of recurrent colitis associated with *Clostridioides difficile* infection was not established until the publication of the first clinical trial in 2013 [75]. In this study, the authors compared the infusion of donor faeces preceded by an abbreviated regimen of vancomycin and bowel lavage, a standard vancomycin regimen, and a standard vancomycin regimen with bowel lavage. The study was stopped after only 43 of the 120 patients were randomised, since the infusion of donor faeces was significantly more effective (81% of the patients) than the use of vancomycin (31% of patients, *p* < 0.001) after the first infusion.

Following these results, the administration of FMT has been further developed. It is now available through different routes, such as nasogastric sonde, enema, administration via colonoscopy, and, more recently, administration in oral capsules.

New indications for FMT have been also investigated. Given the association between gut dysbiosis and the development of IBD, the application of FMT for the management of patients with IBD has been intensively explored. There are some promising results in patients with UC refractory to the standard treatment. However, the studies in this field are limited due to the heterogeneity of the inclusion criteria and the infusion protocols, such as the recruitment of donors, the preparation of faecal material, the selection of the route of administration, or the number of infusions. To address the lack of a regulatory framework, an international panel of experts has proposed consensus-based statements and recommendations based on the available evidence to move towards standardised practices [75]. This first international Rome consensus conference on the gut microbiota and FMT offers clear standardised protocols for donor selection and biobanking, the general organisation and criteria required to promote FMT as a recognised strategy for the treatment of IBD.

This consensus document limits the application of FMT as a therapeutical option for recurrent *C. difficile* infection. The recommended application of FMT in patients with UC is framed to clinical research and not standard clinical practice due to the currently available evidence. There is insufficient evidence to support the use of FMT in patients with CD in clinical practice.

## 5. Potential Microbiome-Based Therapies for Inflammatory Bowel Disease

As knowledge of the gut microbiome advances, new approaches to managing the chronic inflammatory response in IBD are being developed. Although they are still in the preclinical phase, they provide innovative and interesting proposals.

### 5.1. Phagotherapie

Bacteriophages, viruses that selectively infect bacteria, have been investigated for their therapeutic potential, as they can target and eliminate a specific bacterial strain. In a recent proof-of-concept study, it was found that *Klebsiella pneumoniae* strains were strongly associated with IBD and, subsequently, a combination phage therapy was developed to selectively eliminate these strains [56]. The administration of a combination of bacteriophages to mice colonised with the pathogenic *K. pneumoniae* strain attenuated inflammation in a colitis model and was found to be safe and viable in healthy subjects.

Furthermore, there are some interesting data about the imbalance of the gut virome and bacteriophages in patients with *C. difficile* infection. Given the potential risks associated with the transfer of living microorganisms during FMT, such as for infectious, metabolic, or malignant diseases, researchers have investigated the transfer of a sterile faecal filtrate [76]. This filtrate contained bacterial debris, proteins, and antimicrobial and metabolic compounds. The study involved five patients with *C. difficile* infection and, in all patients, the transfer of the sterile faecal filtrate eliminated the symptoms of the disease for a minimum period of 6 months. It was hypothesised that the transfer of bacteriophages in the filtrate could have been involved in the success of the treatment, as all samples were dominated by a rich variety of Lactococcus bacteriophages.

Another study focused on the phageomes of patients who received FMT for the treatment of *C. difficile* infection [77]. The study revealed gut virome dysbiosis in these patients, characterised by an increase in *Caudovirales* abundance and decreased *Caudovirales* diversity, richness, and uniformity compared to healthy controls. The results showed a high bacteriophage transfer ratio during FMT, resulting in a higher colonisation level of *Caudovirales* phage species in the receptor. These results were associated with better FMT efficacy.

### 5.2. Spore-Based Therapy

Firmicutes are bacteria that can form spores. These bacteria are relevant in gut homeostasis through the production of metabolites that can enhance the function of the gastrointestinal barrier and mucosal immune system [78]. The spores are intrinsically resistant to gastric acid. Once they germinate, they become metabolically active bacteria that can colonise the colon. Based on the evidence that Firmicutes bacteria and the associated metabolites are reduced in the gut microbiome in patients with UC, a small phase 1b trial with Firmicutes spores formulated into oral capsules was conducted. The trial found that the oral administration of Firmicutes spores, after vancomycin preconditioning, induced clinical remission in a significantly higher proportion of patients with mild to moderately active UC than a placebo [79]. This clinical trial provided evidence of a favourable safety profile.

### 5.3. Next-Generation Probiotics and Live Biotherapeutics

The field of probiotics has advanced to the development of next-generation probiotics (NGPs) and live biotherapeutic products (LBPs). Unlike traditional probiotics, these products have not been previously tested in humans. Instead, NGPs and LBPs are obtained from the advanced sequencing and bioinformatic analysis of the human gut microbiome and are primarily used to treat or cure disease conditions. In contrast to probiotics, which are typically marketed as dietary food supplements after their evaluation to ensure safety, NGPs are subject to a more strict regulatory framework. Faecalibacterium shows favourable results as an LBP due to its role in host homeostasis and its positive effects in preclinical animal models for several diseases, including IBD [80]. In fact, a phase 1 multicentre study is ongoing to evaluate its safety and preliminary efficacy in patients with mild to moderate CD with a corticosteroid-induced clinical response [81].

In 2004, researchers at the Wageningen Microbiology Laboratory in the Netherlands isolated, for the first time, a strain capable of growing on a viscous substrate, such as mucin, from the guts of healthy adults [82]. It was identified as a new species of the genus Verrucomicrobiota called *Akkermansia muciniphila*, a mucin-degrading bacterium, and represented as strain MucT [82]. For years, the MucT strain of *A. muciniphila* was the only strain isolated from humans, but, in the last 5 years, new strains have been identified. By degrading mucin, *A. muciniphila* produces acetate and propionate, which serve as substrates for other bacteria and the host. Consequently, it promotes mucin turnover and thickening, reinforcing the intestinal barrier and reducing the gut permeability to microbial products. It is considered a biomarker of a healthy host metabolic profile, and the decreased abundance of *A. Muciniphila* is related to several metabolic and inflammatory diseases, such as IBD.

Although it is the most promising NGP, the clinical application of *A. muciniphila* is still limited by several factors. Its role in IBD pathogenesis remains undefined, with studies showing contradictory results [83,84,85,86]. This could be associated with the host genotype, the strain specificity of *A. muciniphila*, or the coexistence of other enteropathogens. Secondly, using live *A. muciniphila* for therapeutic purposes is challenging due to its instability as an anaerobic bacterium. Cultivating and maintaining the bacteria’s stability and viability outside of the gut is difficult. However, the pasteurisation of *A. muciniphila* and its approval as a postbiotic has opened a new research line in managing metabolic diseases and other inflammatory disorders [87]. Thirdly, the balance between different types of bacteria populations in the gut microbiome may determine the susceptibility to inflammation-associated colon tumorigenesis [88]. In some cases, *A. muciniphila* may exacerbate gut inflammation and correlate with higher rates of tumorigenesis [89].

### 5.4. Targeting Altered Microbiome Function: AhR Agonists

Another promising approach is to restore the altered microbiome function. Data have suggested that the end products of tryptophan metabolism are essential to maintain intestinal homeostasis between the mucosal immune system and gut microbiota via AhR by modulating the production of interleukin (IL)-22 [90]. A recent study conducted on a large IBD cohort observed an imbalance in tryptophan metabolism [51]. The researchers explored the metabolites of the kynurenine pathway in mice and humans and observed a link between intestinal inflammation and this metabolic route. Intestinal inflammation correlated negatively with the amounts of xanthurenic (XANA) and kynurenic (KYNA) acids, which are AhR ligands. Supplementation with XANA or KYNA decreased the colitis severity through effects on intestinal epithelial cells and T cells, involving AhR activation [91]. These results set the scene for the use of metabolites derived from the gut microbiota as biomarkers for dysbiosis and/or the targets of new therapeutic interventions in patients with IBD.

### 5.5. Bile Acids

There have been substantial scientific advances in the field of bile acids and the gut microbiome with the discovery of a new pool of bile acid structures and their central role in metabolism and immune homeostasis [92]. This might lead to the manipulation of BAs through the bioengineering of the gut microbiota to modulate the BA pool.

## 6. Discussion

This review aimed to summarise the limited understanding of the role of the human gut microbiome in the development and progression of IBD. The emergence and availability of the new “multi-omics” technologies are expected to facilitate the better characterisation of this dynamic and diverse ecosystem of commensal bacteria, fungi, and viruses.

There are still methodological challenges in studying the human gut microbiota [19]. The research on the IBD microbiome has mostly focused on high-level taxonomic classification. However, to gain a more thorough understanding of the disease and its underlying causes, it is necessary to study the microbiome at a strain level and investigate the functionality of the microorganisms. Additionally, the available reference data required for the use of NGS analyses are mostly limited to North America and Europe, which implies a bias in the study of a disease that has been associated with industrialisation and a Western lifestyle.

Regarding the collection process, most studies of the human gut microbiome have used faecal samples due to practical reasons such as ease of acquisition, reduced costs, and non-invasiveness. However, this method may not provide accurate information about the mucosa-associated microbiome [18]. Mucosal biopsies collected by endoscopy could offer a better representation of this population, although this technique presents other limitations, such as the required fasting and colon cleansing, which could alter the microbiome. Furthermore, it is not clear whether the DNA extraction methods and storage conditions in biobanks affect the collected samples.

One of the main goals of studying the gut microbiome in patients with IBD is to find biomarkers that can identify individuals at risk of developing IBD or those in the early stages of the disease [18]. This could help to determine their prognosis, stratify patients for different therapeutic approaches, or detect patients with a higher risk of relapse. However, there is still limited knowledge about the composition and functionality of the microbiome, especially in the viral and fungal populations. Identifying a biomarker that applies to all patients is a complex task due to the intra- and inter-individual variability of the human gut microbiome. Additionally, the exact role of dysbiosis in the development of IBD is still unclear, and we cannot determine whether it precedes the inflammatory process or is a reflection of the altered immune and metabolic environment [32]. To overcome this challenge, it would be necessary to conduct well-designed longitudinal studies, progressing from searching for correlations to searching for causation in the investigation of the role of the microbiome in IBD.

Research is ongoing to develop microbiome-based interventions for patients with IBD, but there is still no well-defined approach. Currently, efforts are being made to restore a balanced microbiome through dietary modifications, the use of prebiotics and probiotics, and FMT. However, investigations into the effect of the diet on patients with IBD face several limitations, including the duration and timing of exposure to dietary patterns, the methods used to assess dietary intake, and the detection of specific dietary components such as food additives or food processing [54]. In the case of probiotics, the identification of new strains with therapeutic potential will be aided by the development of advanced techniques for the study of the microbiome [75,93]. As for FMT, it aims to restore the diversity of the entire gut microbiome, and although it has shown some positive effects in patients with UC, its use is still limited to research studies due to the many uncertainties related to the mechanism of action and potential long-term effects [75].

Other potential strategies are currently being explored, and bacteriophages are gaining relevance in this field. A recent publication identified changes in metagenomic samples from IBD cohorts that impacted the DNA inversion states and functionality of the gut microbiota, highlighting the correlation of bacteriophages with host inflammation in IBD [94]. However, there is still a long way to go before bacteriophages can be implemented for the treatment of patients with IBD. It is essential to obtain a comprehensive sequencing database and to understand the interactions between bacteriophages and human health. In addition, one crucial aspect that needs to be addressed is whether bacteriophages can activate aberrant immune responses. Genetically engineered probiotics are an exciting prospect for the future treatment of patients with IBD [95]. Better culturing methods, more affordable sequencing techniques, and more powerful tools are needed to facilitate their development. Before implementation, preclinical trials and a strict regulatory process are necessary to explore the safety and toxicity of the products. Lastly, the study of the metabolome and its multiple pathways may offer new therapeutic lines in the future.

In conclusion, a better understanding of the human gut microbiome and its role in the IBD pathogenesis could provide more precise tools for the management of IBD.

## References

[1] De Souza H.S.P., Fiocchi C. (2016). Immunopathogenesis of IBD: Current state of the art. Nat. Rev. Gastroenterol. Hepatol..

[2] Guzzo G.L., Andrews J.M., Weyrich L.S. (2022). The Neglected Gut Microbiome: Fungi, Protozoa, and Bacteriophages in Inflammatory Bowel Disease. Inflamm. Bowel Dis..

[3] Hoffmann C., Dollive S., Grunberg S., Chen J., Li H., Wu G.D., Lewis J.D., Bushman F.D. (2013). Archaea and Fungi of the Human Gut Microbiome: Correlations with Diet and Bacterial Residents. PLoS ONE.

[4] Marchesi J.R., Ravel J. (2015). The vocabulary of microbiome research: A proposal. Microbiome.

[5] Dave M., Higgins P.D., Middha S., Rioux K.P. (2012). The human gut microbiome: Current knowledge, challenges, and future directions. Transl. Res..

[6] Ananthakrishnan A.N. (2015). Epidemiology and risk factors for IBD. Nat. Rev. Gastroenterol. Hepatol..

[7] Turnbaugh P.J., Ley R.E., Hamady M., Fraser-Liggett C.M., Knight R., Gordon J.I. (2007). The Human Microbiome Project. Nature.

[8] Liguori G., Lamas B., Richard M.L., Brandi G., da Costa G., Hoffmann T.W., Di Simone M.P., Calabrese C., Poggioli G., Langella P. (2016). Fungal dysbiosis in mucosa-associated microbiota of Crohn’s disease patients. J. Crohns Colitis.

[9] Sokol H., Leducq V., Aschard H., Pham H.-P., Jegou S., Landman C., Cohen D., Liguori G., Bourrier A., Nion-Larmurier I. (2017). Fungal microbiota dysbiosis in IBD. Gut.

[10] Manrique P., Dills M., Young M.J. (2017). The human gut phage community and its implications for health and disease. Viruses.

[11] Breugelmans T., Van Spaendonk H., De Man J.G., De Schepper H., Jauregui-Amezaga A., Macken E., Lindén S., Pintelon I., Timmermans J.P., De Winter B.Y. (2020). In-depth study of transmembrane mucins in association with intestinal barrier dysfunction during the course of t cell transfer and dss-induced colitis. J. Crohns Colitis.

[12] Breugelmans T., Oosterlinck B., Arras W., Ceuleers H., De Man J., Hold G.L., De Winter B.Y., Smet A. (2022). The role of mucins in gastrointestinal barrier function during health and disease. Lancet Gastroenterol. Hepatol..

[13] Breugelmans T., Arras W., Boen L.E., Borms E., Kamperdijk L., De Man J., Van de Vijver E., Van Gils A., De Winter B.Y., Moes N. (2023). Aberrant Mucin Expression Profiles Associate with Pediatric Inflammatory Bowel Disease Presentation and Activity. Inflamm. Bowel Dis..

[14] Lacroix V., Cassard A., Mas E., Barreau F. (2021). Multi-omics analysis of gut microbiota in inflammatory bowel diseases: What benefits for diagnostic, prognostic and therapeutic tools?. Int. J. Mol. Sci..

[15] Fiocchi C., Dragoni G., Iliopoulos D., Katsanos K., Hernandez Ramirez V., Suzuki K. (2021). Scientific Workshop Steering Committee Results of the Seventh Scientific Workshop of ECCO: Precision Medicine in IBD-What, Why and How. J. Crohns Colitis.

[16] Aldars-García L., Chaparro M., Gisbert J.P. (2021). Systematic review: The gut microbiome and its potential clinical application in inflammatory bowel disease. Microorganisms.

[17] Wensel C.R., Pluznick J.L., Salzberg S.L., Sears C.L. (2022). Next-generation sequencing: Insights to advance clinical investigations of the microbiome. J. Clin. Investig..

[18] Boix-Amorós A., Monaco H., Sambataro E., Clemente J.C. (2022). Novel technologies to characterize and engineer the microbiome in inflammatory bowel disease. Gut Microbes.

[19] Chang J.T. (2020). Pathophysiology of Inflammatory Bowel Diseases. N. Engl. J. Med..

[20] Strachan D.P. (1989). Hay fever, hygiene, and household size. BMJ.

[21] Qin J., Li R., Raes J., Arumugam M., Burgdorf K.S., Manichanh C., Nielsen T., Pons N., Levenez F., Yamada T. (2010). A human gut microbial gene catalogue established by metagenomic sequencing. Nature.

[22] Pittayanon R., Lau J.T., Leontiadis G.I., Tse F., Yuan Y., Surette M., Moayyedi P. (2020). Differences in Gut Microbiota in Patients with vs without Inflammatory Bowel Diseases: A Systematic Review. Gastroenterology.

[23] Sokol H., Pigneur B., Watterlot L., Lakhdari O., Bermúdez-Humarán L.G., Gratadoux J.J., Blugeon S., Bridonneau C., Furet J.P., Corthier G. (2008). Faecalibacterium prausnitzii is an anti-inflammatory commensal bacterium identified by gut microbiota analysis of Crohn disease patients. Proc. Natl. Acad. Sci. USA.

[24] Gophna U., Sommerfeld K., Gophna S., Doolittle W.F., Veldhuyzen Van Zanten S.J.O. (2006). Differences between tissue-associated intestinal microfloras of patients with Crohn’s disease and ulcerative colitis. J. Clin. Microbiol..

[25] Sun S., Zhu X., Huang X., Murff H.J., Ness R.M., Seidner D.L., Sorgen A.A., Blakley I.C., Yu C., Dai Q. (2021). On the robustness of inference of association with the gut microbiota in stool, rectal swab and mucosal tissue samples. Sci. Rep..

[26] Limon J.J., Tang J., Li D., Wolf A.J., Michelsen K.S., Funari V., Gargus M., Nguyen C., Sharma P., Maymi V.I. (2019). Malassezia Is Associated with Crohn’s Disease and Exacerbates Colitis in Mouse Models. Cell Host Microbe.

[27] Cao Z., Fan D., Sun Y., Huang Z., Li Y., Su R., Zhang F., Li Q., Yang H., Zhang F. (2024). The gut ileal mucosal virome is disturbed in patients with Crohn’s disease and exacerbates intestinal inflammation in mice. Nat. Commun..

[28] Thomas J.P., Modos D., Rushbrook S.M., Powell N., Korcsmaros T. (2022). The Emerging Role of Bile Acids in the Pathogenesis of Inflammatory Bowel Disease. Front. Immunol..

[29] Wang S., van Schooten F.J., Jin H., Jonkers D., Godschalk R. (2023). The Involvement of Intestinal Tryptophan Metabolism in Inflammatory Bowel Disease Identified by a Meta-Analysis of the Transcriptome and a Systematic Review of the Metabolome. Nutrients.

[30] Lamas B., Natividad J.M., Sokol H. (2018). Aryl hydrocarbon receptor and intestinal immunity review-article. Mucosal Immunol..

[31] Ni J., Wu G.D., Albenberg L., Tomov V.T. (2017). Gut microbiota and IBD: Causation or correlation?. Nat. Rev. Gastroenterol. Hepatol..

[32] Lavelle A., Sokol H. (2020). Gut microbiota-derived metabolites as key actors in inflammatory bowel disease. Nat. Rev. Gastroenterol. Hepatol..

[33] Clooney A.G., Eckenberger J., Laserna-Mendieta E., Sexton K.A., Bernstein M.T., Vagianos K., Sargent M., Ryan F.J., Moran C., Sheehan D. (2021). Ranking microbiome variance in inflammatory bowel disease: A large longitudinal intercontinental study. Gut.

[34] Atkinson A.J., Colburn W.A., DeGruttola V.G., DeMets D.L., Downing G.J., Hoth D.F., Oates J.A., Peck C.C., Schooley R.T., Spilker B.A. (2001). Biomarkers and surrogate endpoints: Preferred definitions and conceptual framework. Clin. Pharmacol. Ther..

[35] Papa E., Docktor M., Smillie C., Weber S., Preheim S.P., Gevers D., Giannoukos G., Ciulla D., Tabbaa D., Ingram J. (2012). Non-invasive mapping of the gastrointestinal microbiota identifies children with inflammatory bowel disease. PLoS ONE.

[36] Estevinho M.M., Rocha C., Correia L., Lago P., Ministro P., Portela F., Trindade E., Afonso J., Peyrin-Biroulet L., Magro F. (2020). Features of Fecal and Colon Microbiomes Associate with Responses to Biologic Therapies for Inflammatory Bowel Diseases: A Systematic Review. Clin. Gastroenterol. Hepatol..

[37] Vich Vila A., Imhann F., Collij V., Jankipersadsing S.A., Gurry T., Mujagic Z., Kurilshikov A., Bonder M.J., Jiang X., Tigchelaar E.F. (2018). Gut microbiota composition and functional changes in inflammatory bowel disease and irritable bowel syndrome. Sci. Transl. Med..

[38] Ryan F.J., Ahern A.M., Fitzgerald R.S., Laserna-Mendieta E.J., Power E.M., Clooney A.G., O’Donoghue K.W., McMurdie P.J., Iwai S., Crits-Christoph A. (2020). Colonic microbiota is associated with inflammation and host epigenomic alterations in inflammatory bowel disease. Nat. Commun..

[39] Turner D., Ricciuto A., Lewis A., D’Amico F., Dhaliwal, Griffiths A.M., Bettenworth D., Sandborn W.J., Sands B.E., Reinisch W. (2021). STRIDE-II: An Update on the Selecting Therapeutic Targets in Inflammatory Bowel Disease (STRIDE) Initiative of the International Organization for the Study of IBD (IOIBD): Determining Therapeutic Goals for Treat-to-Target strategies in IBD. Gastroenterology.

[40] Torres J., Bonovas S., Doherty G., Kucharzik T., Gisbert J.P., Raine T., Adamina M., Armuzzi A., Bachmann O., Bager P. (2020). ECCO guidelines on therapeutics in Crohn’s disease: Medical treatment. J. Crohns Colitis.

[41] Raine T., Bonovas S., Burisch J., Kucharzik T., Adamina M., Annese V., Bachmann O., Bettenworth D., Chaparro M., Czuber-Dochan W. (2022). ECCO Guidelines on Therapeutics in Ulcerative Colitis: Medical Treatment. J. Crohns Colitis.

[42] Spinelli A., Bonovas S., Burisch J., Kucharzik T., Adamina M., Annese V., Bachmann O., Bettenworth D., Chaparro M., Czuber-Dochan W. (2022). ECCO Guidelines on Therapeutics in Ulcerative Colitis: Surgical Treatment. J. Crohns Colitis.

[43] Van Rheenen P.F., Aloi M., Assa A., Bronsky J., Escher J.C., Fagerberg U.L., Gasparetto M., Gerasimidis K., Griffiths A., Henderson P. (2021). The Medical Management of Paediatric Crohn’s Disease: An ECCO-ESPGHAN Guideline Update. J. Crohns Colitis.

[44] Zhang L., Agrawal M., Ng S.C., Jess T. (2024). Early-life exposures and the microbiome: Implications for IBD prevention. Gut.

[45] Raman M., Rajagopalan V., Kaur S., Reimer R.A., Ma C., Ghosh S., Vallance J. (2022). Physical Activity in Patients with Inflammatory Bowel Disease: A Narrative Review. Inflamm. Bowel Dis..

[46] Borrelli O., Cordischi L., Cirulli M., Paganelli M., Labalestra V., Uccini S., Russo P.M., Cucchiara S. (2006). Polymeric Diet Alone Versus Corticosteroids in the Treatment of Active Pediatric Crohn’s Disease: A Randomized Controlled Open-Label Trial. Clin. Gastroenterol. Hepatol..

[47] Narula N., Dhillon A., Zhang D., Sherlock M.E., Tondeur M., Zachos M. (2018). Enteral nutritional therapy for induction of remission in Crohn’s disease. Cochrane Database Syst. Rev..

[48] Gatti S., Galeazzi T., Franceschini E., Annibali R., Albano V., Verma A.K., De Angelis M., Lionetti M.E., Catassi C. (2017). Effects of the exclusive enteral nutrition on the microbiota profile of patients with crohn’s disease: A systematic review. Nutrients.

[49] Pigneur B., Lepage P., Mondot S., Schmitz J., Goulet O., Doré J., Ruemmele F.M. (2019). Mucosal Healing and Bacterial Composition in Response to Enteral Nutrition Vs Steroid-based Induction Therapy—A Randomised Prospective Clinical Trial in Children with Crohn’s Disease. J. Crohns Colitis.

[50] Lamas B., Richard M.L., Leducq V., Pham H.P., Michel M.L., Da Costa G., Bridonneau C., Jegou S., Hoffmann T.W., Natividad J.M. (2016). ARD9 impacts colitis by altering gut microbiota metabolism of tryptophan into aryl hydrocarbon receptor ligands. Nat. Med..

[51] Wedrychowicz A., Kowalska-Duplaga K., Jedynak-Wasowicz U., Pieczarkowski S., Sladek M., Tomasik P., Fyderek K. (2011). Serum concentrations of VEGF and TGF-β1 during exclusive enteral nutrition in IBD. J. Pediatr. Gastroenterol. Nutr..

[52] Levine A., Wine E., Assa A., Sigall Boneh R., Shaoul R., Kori M., Cohen S., Peleg S., Shamaly H., On A. (2019). Crohn’s Disease Exclusion Diet Plus Partial Enteral Nutrition Induces Sustained Remission in a Randomized Controlled Trial. Gastroenterology.

[53] Limketkai B.N., Godoy-Brewer G., Parian A.M., Noorian S., Krishna M., Shah N.D., White J., Mullin G.E. (2023). Dietary Interventions for the Treatment of Inflammatory Bowel Diseases: An Updated Systematic Review and Meta-analysis. Clin. Gastroenterol. Hepatol..

[54] Nieva C., Pryor J., Williams G.M., Hoedt E.C., Burns G.L., Eslick G.D., Talley N.J., Duncanson K., Keely S. (2024). The Impact of Dietary Interventions on the Microbiota in Inflammatory Bowel Disease: A Systematic Review. JCC.

[55] Federici S., Kredo-Russo S., Valdés-Mas R., Kviatcovsky D., Weinstock E., Matiuhin Y., Silberberg Y., Atarashi K., Furuichi M., Oka A. (2022). Targeted suppression of human IBD-associated gut microbiota commensals by phage consortia for treatment of intestinal inflammation. Cell.

[56] Lo C.H., Khandpur N., Rossato S.L., Lochhead P., Lopes E.W., Burke K.E., Richter J.M., Song M., Ardisson Korat A.V., Sun Q. (2022). Ultra-processed Foods and Risk of Crohn’s Disease and Ulcerative Colitis: A Prospective Cohort Study. Clin. Gastroenterol. Hepatol..

[57] Narula N., Wong E.C.L., Dehghan M., Mente A., Rangarajan S., Lanas F., Lopez-Jaramillo P., Rohatgi P., Lakshmi P.V.M., Varma R.P. (2021). Association of ultra-processed food intake with risk of inflammatory bowel disease: Prospective cohort study. BMJ.

[58] Logan M., Gkikas K., Svolos V., Nichols B., Milling S., Gaya D.R., Seenan J.P., Macdonald J., Hansen R., Ijaz U.Z. (2020). Analysis of 61 exclusive enteral nutrition formulas used in the management of active Crohn’s disease—New insights into dietary disease triggers. Aliment. Pharmacol. Ther..

[59] Fenneman A.C., Weidner M., Chen L.A., Nieuwdorp M., Blaser M.J. (2023). Antibiotics in the pathogenesis of diabetes and inflammatory diseases of the gastrointestinal tract. Nat. Rev. Gastroenterol. Hepatol..

[60] Kronman M.P., Zaoutis T.E., Haynes K., Feng R., Coffin S.E. (2012). Antibiotic exposure and IBD development among children: A population-based cohort study. Pediatrics.

[61] Hviid A., Svanström H., Frisch M. (2011). Antibiotic use and inflammatory bowel diseases in childhood. Gut.

[62] Townsend C.M., Parker C.E., MacDonald J.K., Nguyen T.M., Jairath V., Feagan B.G., Khanna R. (2019). Antibiotics for induction and maintenance of remission in Crohn’s disease. Cochrane Database Syst. Rev..

[63] Gordon M., Sinopoulou V., Grafton-Clarke C., Akobeng A.K. (2022). Antibiotics for the induction and maintenance of remission in ulcerative colitis. Cochrane Database Syst. Rev..

[64] Verburgt C.M., Heutink W.P., Kuilboer L.I.M., Dickmann J.D., van Etten-Jamaludin F.S., Benninga M.A., de Jonge W.J., Van Limbergen J.E., Tabbers M.M. (2021). Antibiotics in pediatric inflammatory bowel diseases: A systematic review. Expert. Rev. Gastroenterol. Hepatol..

[65] Bischoff S.C., Bager P., Escher J., Forbes A., Hébuterne X., Hvas C.L., Joly F., Klek S., Krznaric Z., Ockenga J. (2023). ESPEN guideline on Clinical Nutrition in inflammatory bowel disease. Clin. Nutr..

[66] Guarner F., Sanders M.E., Szajewska H., Cohen H.M., Eliakim R., Herrera-Deguise C., Karakan T., Merenstein D., Piscoya A.M., Ramakrishna B. (2024). World Gastroenterology Organisation Global Guidelines: Probiotics and Prebiotics. J. Clin. Gastroenterol..

[67] Fontana L., Bermudez-Brito M., Plaza Diaz J., Muñoz-Quezada S., Gil A. (2013). Sources, isolation, characterisation and evaluation of probiotics. Br. J. Nutr..

[68] Limketkai B.N., Akobeng A.K., Gordon M., Adepoju A.A. (2020). Probiotics for induction of remission in Crohn’s disease. Cochrane Database Syst. Rev..

[69] Ganji-Arjenaki M., Rafieian-Kopaei M. (2018). Probiotics are a good choice in remission of inflammatory bowel diseases: A meta analysis and systematic review. J. Cell Physiol..

[70] Zhang X.F., Guan X.X., Tang Y.J., Sun J.F., Wang X.K., Wang W.D., Fan J.M. (2021). Clinical effects and gut microbiota changes of using probiotics, prebiotics or synbiotics in inflammatory bowel disease: A systematic review and meta-analysis. Eur. J. Nutr..

[71] Salminen S., Collado M.C., Endo A., Hill C., Lebeer S., Quigley E.M.M., Sanders M.E., Shamir R., Swann J.R., Szajewska H. (2021). The International Scientific Association of Probiotics and Prebiotics (ISAPP) consensus statement on the definition and scope of postbiotics. Nat. Rev. Gastroenterol. Hepatol..

[72] Van Nood E., Vrieze A., Nieuwdorp M., Fuentes S., Zoetendal E.G., de Vos W.M., Visser C.E., Kuijper E.J., Bartelsman J.F., Tijssen J.G. (2013). Duodenal Infusion of Donor Feces for Recurrent Clostridium difficile. N. Engl. J. Med..

[73] Eiseman B., Silen W., Bascom G.S., Kauvar A.J. (1958). Fecal enema as an adjunct in the treatment of pseudomembranous. Surgery.

[74] Lopetuso L.R., Deleu S., Godny L., Petito V., Puca P., Facciotti F., Sokol H., Ianiro G., Masucci L., Abreu M. (2023). The first international Rome consensus conference on gut microbiota and faecal microbiota transplantation in inflammatory bowel disease. Gut.

[75] Ott S.J., Waetzig G.H., Rehman A., Moltzau-Anderson J., Bharti R., Grasis J.A., Cassidy L., Tholey A., Fickenscher H., Seegert D. (2017). Efficacy of Sterile Fecal Filtrate Transfer for Treating Patients with Clostridium difficile Infection. Gastroenterology.

[76] Zuo T., Wong S.H., Lam K., Lui R., Cheung K., Tang W., Ching J.Y.L., Chan P.K.S., Chan M.C.W., Wu J.C.Y. (2018). Bacteriophage transfer during faecal microbiota transplantation in Clostridium difficile infection is associated with treatment outcome. Gut.

[77] Kostic A.D., Xavier R.J., Gevers D. (2014). The microbiome in inflammatory bowel disease: Current status and the future ahead. Gastroenterology.

[78] Henn M.R., O’Brien E.J., Diao L., Feagan B.G., Sandborn W.J., Huttenhower C., Wortman J.R., McGovern B.H., Wang-Weigand S., Lichter D.I. (2021). A Phase 1b Safety Study of SER-287, a Spore-Based Microbiome Therapeutic, for Active Mild to Moderate Ulcerative Colitis. Gastroenterology.

[79] Martín R., Miquel S., Benevides L., Bridonneau C., Robert V., Hudault S., Chain F., Berteau O., Azevedo V., Chatel J.M. (2017). Functional characterization of novel Faecalibacterium prausnitzii strains isolated from healthy volunteers: A step forward in the use of F. prausnitzii as a next-generation probiotic. Front. Microbiol..

[80] Clinical Trial: EXL01 in the Maintenance of Steroid-Induced Clinical Response/Remission in Participants with Mild to Moderate Crohn’s Disease (MAINTAIN). https://clinicaltrials.gov/study/NCT05542355.

[81] Derrien M., Vaughan E.E., Plugge C.M., de Vos W.M. (2004). Akkermansia municiphila gen. nov., sp. nov., a human intestinal mucin-degrading bacterium. Int. J. Syst. Evol. Microbiol..

[82] Png C.W., Lindén S.K., Gilshenan K.S., Zoetendal E.G., McSweeney C.S., Sly L.I., McGuckin M.A., Florin T.H.J. (2010). Mucolytic bacteria with increased prevalence in IBD mucosa augment in vitro utilization of mucin by other bacteria. Am. J. Gastroenterol..

[83] Earley H., Lennon G., Balfe Á., Coffey J.C., Winter D.C., O’Connell P.R. (2019). The abundance of Akkermansia muciniphila and its relationship with sulphated colonic mucins in health and ulcerative colitis. Sci. Rep..

[84] Vigsnæs L.K., Brynskov J., Steenholdt C., Wilcks A., Licht T.R. (2012). Gram-negative bacteria account for main differences between faecal microbiota from patients with ulcerative colitis and healthy controls. Benef. Microbes.

[85] Lopez-Siles M., Enrich-Capó N., Aldeguer X., Sabat-Mir M., Duncan S.H., Garcia-Gil J., Martinez-Medina M. (2018). Alterations in the Abundance and Co-occurrence of Akkermansia muciniphila and Faecalibacterium prausnitzii in the Colonic Mucosa of Inflammatory Bowel Disease Subjects. Front. Cell Infect. Microbiol..

[86] EFSA (European Food Safety Authority) Safety of Pasteurised Akkermansia Muciniphila as a Novel Food Pursuant to Regulation (EU) 2015/2283. https://www.efsa.europa.eu/en/efsajournal/pub/6780.

[87] Baxter N.T., Zackular J.P., Chen G.Y., Schloss P.D. (2014). Structure of the gut microbiome following colonization with human feces determines colonic tumor burden. Microbiome.

[88] Ganesh B.P., Klopfleisch R., Loh G., Blaut M. (2013). Commensal Akkermansia muciniphila Exacerbates Gut Inflammation in Salmonella Typhimurium-Infected Gnotobiotic Mice. PLoS ONE.

[89] Zelante T., Iannitti R.G., Cunha C., De Luca A., Giovannini G., Pieraccini G., Zecchi R., D’Angelo C., Massi-Benedetti C., Fallarino F. (2013). Tryptophan catabolites from microbiota engage aryl hydrocarbon receptor and balance mucosal reactivity via interleukin-22. Immunity.

[90] Michaudel C., Danne C., Agus A., Magniez A., Aucouturier A., Spatz M., Lefevre A., Kirchgesner J., Rolhion N., Wang Y. (2023). Rewiring the altered tryptophan metabolism as a novel therapeutic strategy in inflammatory bowel diseases. Gut.

[91] Mohanty I., Allaband C., Mannochio-Russo H., El Abiead Y., Hagey L.R., Knight R., Dorrestein P.C. (2024). The changing metabolic landscape of bile acids—Keys to metabolism and immune regulation. Nat. Rev. Gastroenterol. Hepatol..

[92] Xu M., Zhang W., Lin B., Lei Y., Zhang Y., Chen B., Mao Q., Kim J.J., Cao Q. (2024). Efficacy of probiotic supplementation and impact on fecal microbiota in patients with inflammatory bowel disease: A systematic review and meta-analysis of randomized controlled trials. Nutr. Rev..

[93] Carasso S., Zaatry R., Hajjo H., Kadosh-Kariti D., Ben-Assa N., Naddaf R., Mandelbaum N., Pressman S., Chowers Y., Gefen T. (2024). Inflammation and bacteriophages affect DNA inversion states and functionality of the gut microbiota. Cell Host Microbe.

[94] Zhang T., Zhang J., Duan L. (2023). The Role of Genetically Engineered Probiotics for Treatment of Inflammatory Bowel Disease: A Systematic Review. Nutrients.

[95] Agus A., Planchais J., Sokol H. (2018). Gut Microbiota Regulation of Tryptophan Metabolism in Health and Disease. Cell Host Microbe.

